# Supplementary material for: Real-world datasets for the International Registry for Alzheimer's Disease and Other Dementias (InRAD) and other registries: An international consensus
Source: J Prev Alzheimers Dis. 2025 Feb 18;12(4):100096. doi: 10.1016/j.tjpad.2025.100096 (PMC12183950; doi:10.1016/j.tjpad.2025.100096)
Supplement: Supplementary file 1 [file mmc1.docx]

**Supplemental material:**

**Perneczky R et al. Real-world datasets for the International Registry for Alzheimer’s Disease and Other Dementias (InRAD) and other registries: an international consensus**

**Supplemental table 1.** Domains identified by the International Steering Committee and items identified for both the minimum dataset and extended datasets, including their frequency of recording (entry visit vs all visits)

The International Steering Committee and consultation process resulted in consensus datasets with domains classified into the following ten groups: (1) patient profile and demographics; (2) Lifestyle and anthropometrics; (3) Co-morbidities and diagnostics; (4) Imaging; (5) Treatment; (6) Clinical characterization; (7) Safety; (8) Discontinuation; (9) Laboratory tests; and (10) Patient and care partner outcomes.

|  | **Minimum Data Set** | | **Extended Data Set** | |
| --- | --- | --- | --- | --- |
|  | **Domain** | **Frequency** | **Domain** | **Frequency** |
| **Patient profile and demographics** | Patient ID | Entry only | Other ID (for data linkage) | Entry only |
|  | Consent | Entry only | Care partner and patient consent for care partner to provide data | Entry and visits |
|  | Care partner and availability as informant | Entry and visits | Relationship to caregiver (spouse, partner, son or daughter, sibling, other relative, other [specify]) | Entry and visits |
|  | Sex assigned at birth | Entry only | Caregiver living with patient | Entry and visits |
|  | Birth year or date of birth | Entry only | Marital Status | Entry |
|  | Race or ethnicity | Entry only | Linguistics: Monolingual, bilingual or multilingual | Entry only |
|  | Country of birth and residence | Entry only | Dominant hand (left, right, ambidextrous) | Entry only |
|  | Education* | Entry only | Work status and current or last occupation | Entry and visits |
|  | Living status (alone, with family or partner, in care) | Entry and visits | Urbanicity of residence (city, town, rural) | Entry and visits |
| **Lifestyle and anthropometrics** | Height | Entry only | Alcohol consumption (units or standardized drinks per week) | Entry and visits |
|  | Weight | Entry and visits | Smoking (pack-years, current, ex-smoker, never smoker) | Entry and visits |
|  |  | | Cannabis and recreational drug use (drug[s], unknown, never, past, current) | Entry and visits |
|  |  |  | Physical activity (hours per week) | Entry and visits |
|  |  |  | Actively driving (date if stopped due to dementia) | Entry and visits |
|  |  |  | Sleep (hours per night; self and/or carer reports or using wearable sleep tracker) | Entry and visits |
| **Diagnostic work-up** | Family History of dementia first-degree relative/diagnosis | Entry only | Clinical specialty making the diagnosis | Entry only |
|  | Date of symptom onset | Entry only | Diagnosis Referral pathway (direct, primary care, other specialty)  Diagnosis specialty: (primary care, neurology, psychiatry, care of the elderly, other) | Entry only |
|  | Diagnosis (Alzheimer’s disease and/or co-morbidity or alternative diagnoses, including separate field for syndromic presentation without diagnosis) | Entry only | Diagnostic evidence of neurodegeneration Neurofilament light chain levels (yes, no, indeterminant, not performed) | Entry and visits |
|  | Predominant AD syndrome/variant in first 2 years (amnestic, posterior, logopenic or other) | Entry only | Previous or current dementia-related clinical trial (phase, dates, medication; entry only) | Entry and visits |
|  | Diagnostic Amyloid β status (yes, no, indeterminant, not performed) | Entry only |  | |
|  | Diagnostic Tau positivity status (yes, no, indeterminant, not performed) | Entry only |  |  |
|  | Diagnostic Imaging evidence of neurodegeneration (yes, no, indeterminant, not performed) | Entry only |  |  |
|  | Diagnostic imaging evidence of pathology secondary to AD (cerebral vascular lesions, cerebral amyloid angiopathy, other) | Entry only |  |  |
|  | Diagnostic biomarker evidence of neurodegeneration, such as neurofilament light and glial fibrillary acidic protein (yes, no, indeterminant, not performed) | Entry only |  |  |
|  | Relevant medical conditions** (history [entry only] and concomitant (entry and visits) | History: entry only  Concomitant: entry and visits |  |  |
| **Imaging** | Imaging (yes, no, date of scan) Type of scan (MRI/CT/PET/DAT) and reason (screening or diagnosis, monitoring progression and/or safety [eg ARIA]), other | Entry and visits | Scanner, magnet strength, Tracer used (eg fluorodeoxyglucose [FDG] Positron emission tomography) | When performed |
|  |  | | PET Quantification (for amyloid β and tau including post-treatment amyloid β quantification) (tracer and centiloid measure) | When performed |
|  |  |  | MRI/CT Atrophy (regional and global) and white matter lesion assessment and other findings (macro-hemorrhage; microhemorrhage; Superficial siderosis; Aria other) | When performed |
|  |  |  | Link to standard accepted minimum radiology report or quantification (yes, no) | When performed |
|  |  |  | Dopamine active transporter (DAT) scan | When performed |
|  |  |  | Laboratory amyloid β levels (CSF/Blood) | Entry and visits |
| **Laboratory tests (blood or cerebrospinal fluid)** | None | | Laboratory tau levels (CSF/Blood) | Entry and visits |
|  |  |  | Neurofilament light chain levels  GFAP | Entry and visits  Entry and visits |
|  |  |  | Biomaterial storage Y/N | Entry and visits |
|  |  |  | Genotype: apolipoprotein E status (note may be minimum for treatment screening) | Entry only |
|  |  |  | Genotype: other mutations and polymorphisms (*APP*, *PSY1*, *PSY2*) | Entry only |
|  |  |  | Complete blood count | When performed |
|  |  |  | Blood chemistry | When performed |
| **Treatment** | AD specific treatments (start/stop date, dose, unit, route and frequency) and reason for discontinuation or change ^¶^ | Entry and visits | Start/Change or stop date | Visits |
|  | Cognitive treatments of interest (entry and visits); including acetylcholinesterase inhibitors; partial antagonists of N-methyl-D-aspartate receptor (memantine); nutritional supplements; others | Entry and visits | Start/Change or stop date | Visits |
|  | Other treatments of interest^§^ | Entry and visits | Clinical Dementia Rating sum of boxes (CDR-SB) global score | Visits |
| **Clinical outcomes** | Global clinical staging (NIA-AA staging) | Entry and visits | Functional Activities Questionnaire (FAQ) score | When used |
|  | Cognitive screening test (yes, no; test MoCA or MMSE Score/version if applicable; | Entry and visits |  |  |
|  | Functional test (yes, no, name of test) | Entry and visits | Amsterdam Instrumental Activities of Daily Living Questionnaire (A-IADL-Q) score | When used |
|  | Milestone events other than Entry and visits  occupation and driving  (eg healthcare service use  and dependence); Has work,  driving and/or work status  changed since the last visit | | Neuropsychiatric Inventory Questionnaire (NPI*-*Q) | When used |
|  |  |  | Utilization in Dementia*-*Lite (RUD-Lite) score | When used |
|  |  |  | Medical events of interest other than those in the MDS (yes, no) | Visits only |
| **Safety** | Serious adverse event^†^ (Event since last visit yes, no and details including dates, MEDdra code, severity and outcome) | Visits only | Outcomes (eg hospitalization, out-patient, rehabilitation, treatment regimen modification) | Visits only |
|  | Amyloid-related imaging abnormalities (Event since last visit yes, no, not performed) | Visits only | Outcomes (eg hospitalization, out-patient, rehabilitation, treatment regimen modification) | When ARIA detected or DMT changed because of tolerability |
|  | Infusion/injection reactions (Event since last visit yes, no, details) | Visits only |  |  |
|  | Other medical events of interest^‡^ (Event since last visit yes, no, details) malignancy, non-ARIA related neurological conditions, serious infections | Visits only |  |  |
| **Patient and care partner outcomes** | None | | Quality of Life in Alzheimer's Disease scale (QoL*-*AD) score | When used |
|  |  |  | Euro-QoL 5 dimensions 5 levels (EQ-5D-5L) score | When used |
|  |  |  | Resource Utilization in Dementia*-*Lite (RUD-Lite) score | When used |
|  |  |  | Dependence Scale score | When used |
|  |  |  | Zarit Burden Interview score | When used |
|  |  |  | Neuropsychiatric Inventory Questionnaire (NPI*-*Q*)* | When used |
|  |  |  | Alzheimer's Disease Cooperative Study - Activities of Daily Living Scale for use in Mild Cognitive Impairment (ADSC_ADL-MCI) |  |
|  |  |  | After final visit | Clinical trial participation and/or eligibility |
| **Discontinuation** | Reason for discontinuation (including death) | After final visit |  |  |

* Using the International Standard Classification of Education [13].

** Cerebrovascular and other vascular or cardiac disorders, psychiatric conditions, other neurological/neurodegenerative conditions (including traumatic brain injury) as well as other significant other historic or on-going conditions including metabolic conditions (eg diabetes, obesity), malignancies, blood or lymphatic diseases, immune or autoimmune conditions, or history of severe or recurrent infections.

† Death, is life-threatening, permanent/serious disability or incapacity, requires/prolongs hospitalization, congenital anomaly/birth defect, other medical important condition [39].

‡ Including serious malignancy, serious infections, other non-ARIA related neurological conditions.

§ Antidepressants, anxiolytics, antipsychotics, antiseizure medications, sedatives, sleep aids (including continuous positive airway pressure), anti-diabetic and weight loss medication, cardiovascular medicines (eg antihypertensives, antilipidemic, antiplatelet, anticoagulants)

¶ Treatment-related adverse reaction or tolerability; lack of effectiveness or disease progression; patient or caregiver choice; scheduled stop or end of treatment; other (eg poor adherence) specified in a free text box.
